# Restoring Wrist Harmony: A Case Report of Ulnar Osteotomy for Correcting the Radius Malunion

**DOI:** 10.7759/cureus.48771

**Published:** 2023-11-14

**Authors:** Tushar Chaudhari, Sushant Kumar, Mukesh O Phalak, Anteshwar Birajdar, Archit Gupta

**Affiliations:** 1 Orthopaedics, Dr. D. Y. Patil Medical College, Hospital and Research Centre, Pune, IND

**Keywords:** distal radial fracture, madelung’s deformity, malunion, positive ulnar variance, ulnar osteotomy, malunited

## Abstract

A 41-year-old female patient sought medical attention due to a malunited distal radius fracture with a positive ulnar variance, experiencing wrist pain and limited range of motion. The patient was successfully treated with an isolated ulnar osteotomy and bone grafting, resulting in significant alleviation of symptoms and improved wrist mobility. Various surgical methods have been proposed to address malunited radius fractures, and ulnar osteotomy has shown promise as an effective technique for such cases.

## Introduction

Fractures of the distal radius are among the most common orthopaedic injuries, often resulting from trauma or falls. While many of these fractures heal with appropriate treatment, malunion, or imperfect healing, can lead to significant functional impairment and discomfort in affected individuals. One surgical technique that has gained recognition and acceptance in addressing malunited distal radius fractures is ulnar osteotomy.

Ulnar osteotomy involves the surgical manipulation and realignment of the ulna bone to restore the proper anatomical relationship between the ulna and radius. This procedure aims to correct the deformities and functional deficits caused by malunited distal radius fractures, ultimately improving the patient's quality of life and wrist function.

In this case report, we present a detailed analysis of a patient who underwent ulnar osteotomy for the correction of a malunited distal radius fracture. We will explore the patient's clinical history, radiographic findings, surgical technique, postoperative outcomes, and the overall effectiveness of ulnar osteotomy as a therapeutic intervention for this specific type of wrist injury. By examining this case, we aim to contribute valuable insights into the management of malunited distal radius fractures and shed light on the potential benefits of ulnar osteotomy in restoring wrist function and relieving patient discomfort.

## Case presentation

History

**Figure 1:**
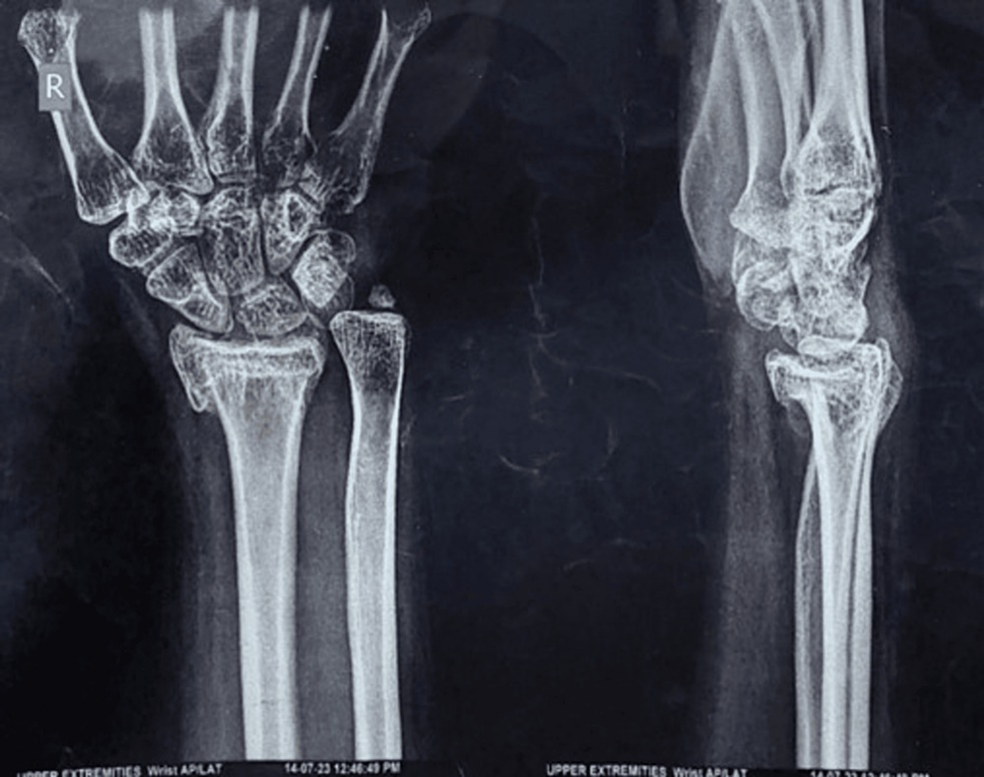
Pre-operative radiograph

A 41-year-old female presented with complaints of pain in the left wrist for six months. The patient had a history of slip and fall six months ago, which was diagnosed as a distal end-of-radius fracture on a plain radiograph. The fracture was managed conservatively in a cast for six weeks and the post-cast removal X-ray (Figure 1) showed an impacted distal end of the radius. The patient was given analgesics and started on wrist range of movement physiotherapy but the patient reported no improvement in symptoms.

**Figure 2:**
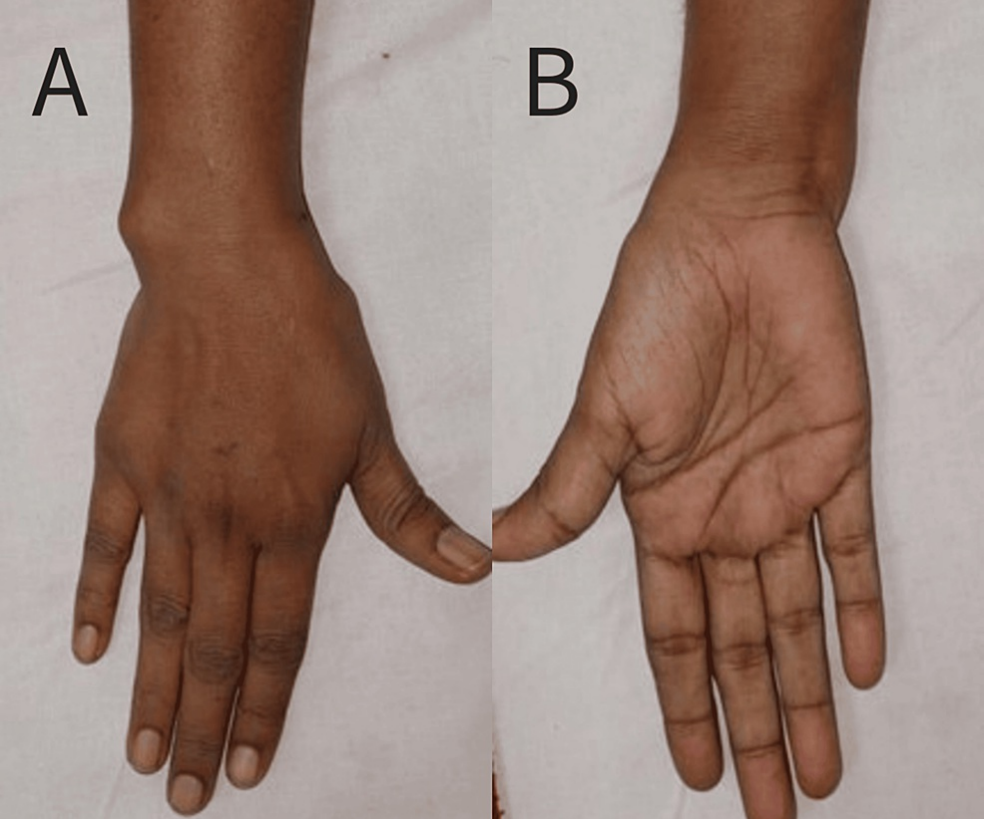
Pre-operative clinical photograph A - Dorsal aspect B - Volar aspect

The patient came to us with complaints of restricted wrist range of motion and pain. There was a deformity showing positive ulnar variance (Figure 2). The patient was advised corrective osteotomy for this and underwent an isolated ulnar osteotomy with plating and bone grafting.

Procedure

The patient underwent a supraclavicular block and was positioned in a supine manner with a sidearm board. The surgical procedure began with the preparation, which included scrubbing, painting, and draping. A standard approach to the ulna was employed, involving a 7 cm incision. Sequentially, both superficial and deep dissections were performed.

**Figure 3:**
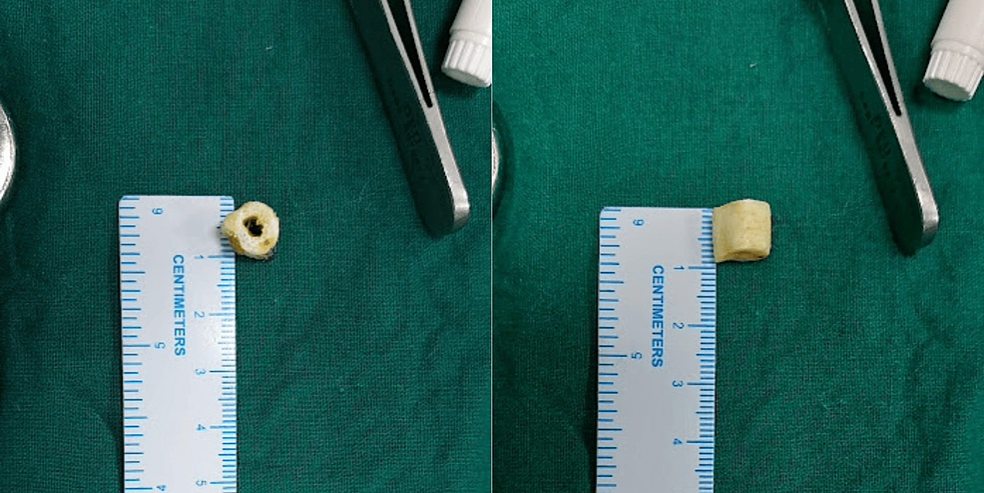
A size of 1x1 cm piece of ulna that was removed

**Figure 4:**
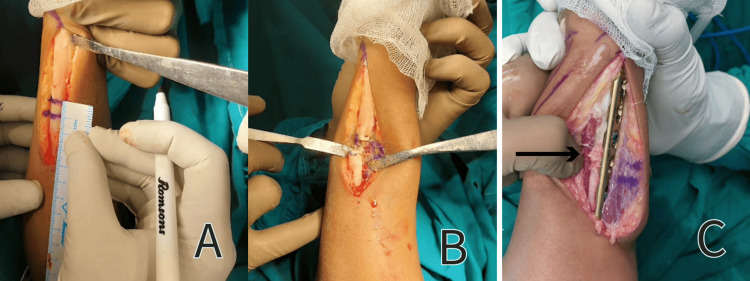
Surgical procedure A - Marking the site for osteotomy B - After removing a 1x1cm piece from the ulna C - Bone graft (arrow)

A critical step in the procedure involved marking the ulna shaft at a point 4 cm from the ulnar styloid. Subsequently, two parallel cuts were made using a bone saw, resulting in the removal of a 1 cm long bone fragment (Figure 3). The ends of the ulna were then secured with bone-holding forceps and brought into alignment. To stabilize the fractured site, a six-hole dynamic compression plate was affixed to the distal fragment using cortical screws. In order to produce compression at the fracture site, an eccentrically positioned cortical screw was also used in the proximal fragment (Figure 4).

To verify the accuracy of the reduction, imaging with a C-arm was performed before securing the reduction with locking screws. A bone graft, harvested from the radial metaphysis at the distal end, was interposed at the fracture site. The closure of the surgical site was conducted in layers, followed by the application of an aseptic sterile dressing. Finally, the patient's arm was immobilized in an above-elbow slab with the forearm in a supinated position.

Results

**Figure 5:**
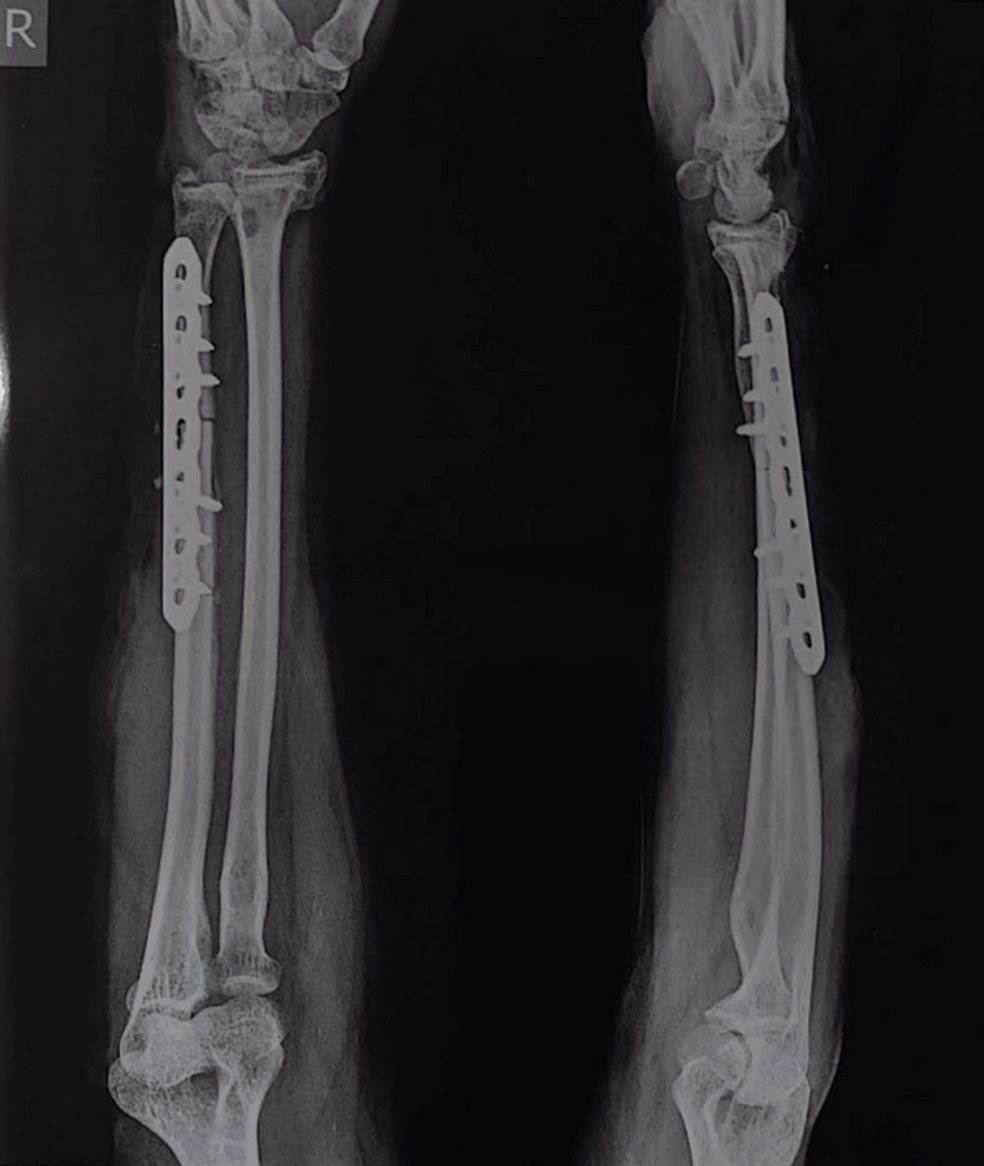
Post-operative radiograph

**Figure 6:**
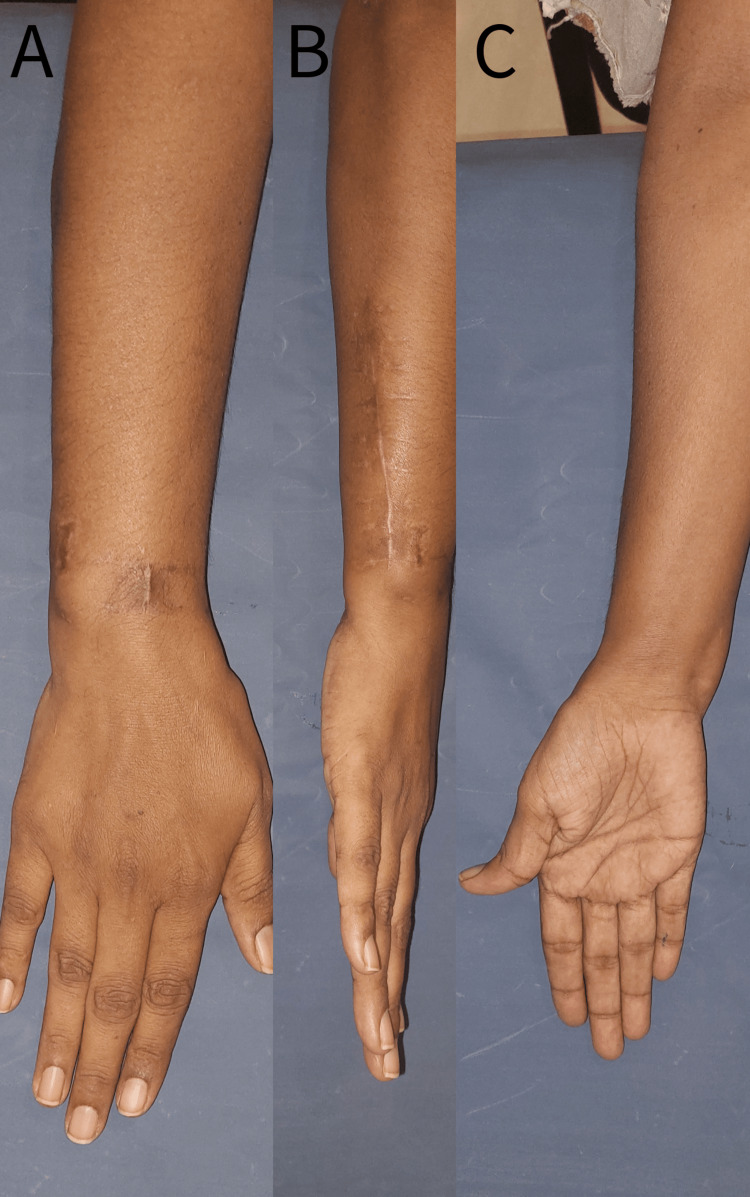
Post-operative clinical photographs

The post-operative radiograph showed a correction of positive ulnar variance (Figure 5). The range of motion in flexion and extension increased from 78° before the surgery to 104° after the surgery. Similarly, the pronation and supination range improved from 120° before the surgery to 163° after the surgery. Additionally, the patient's pain, as assessed by a visual analogue scale, decreased from a score of 9 before the surgery to 3 after the surgery (Figure 6).

## Discussion

Ulnar shortening osteotomy (USO) was initially introduced by Milch as a method to address radial shortening following the distal end of radius fractures [1]. About 3% of all fractures in the upper extremity are distal end-of-radius fractures, making it one of the most frequent upper extremity injuries. When these fractures are not properly managed, they can lead to radial shortening as the primary deformity, which may result in disturbances in radioulnar variance, issues with the triangular fibrocartilage complex (TFCC), and problems with the distal radioulnar joint (DRUJ).

These complications can culminate in ulnar impaction syndrome due to malunion, resulting in symptoms such as pain, restricted motion, reduced pinch and grip strength, and eventually various degrees of osteoarthritis and functional limitations. There are several surgical approaches available to manage extra-articular malunions of the distal radius causing ulnar impaction syndrome such as corrective USO and distal radius osteotomy (DRO).

Surgery to treat ulnar impaction syndrome brought on by distal radius malunions primarily aims to rectify the length connection between the ulna and radius without significantly affecting the joint surfaces or altering volar tilt or radial inclination. DRO has shown encouraging results, especially in instances with painful angulations and malunions greater than 25°. However, DRO is a technically demanding procedure that often entails a lengthy operative time and may require additional steps like bone grafting and concurrent ulnar osteotomies.

In contrast to earlier studies of DRO, Srinivasan and colleagues [2] recently provided evidence of shorter operating times and lower complication rates when treating distal radius malunions with ulnar impaction syndrome with isolated USO.

The DRUJ is classified as a diarthrodial joint, characterized by the larger curvature radius of the concave surface of the sigmoid notch. This unique anatomical feature allows the radiocarpal unit to exhibit both rotational and translational movements around the relatively stable ulna [3,4]. The morphology of the sigmoid notch can vary in the coronal plane, presenting as a flat surface, a sloping configuration, or adopting C or S shapes [5]. The impact of these morphological variations on post-injury outcomes and treatment decisions remains somewhat unclear, but a deeper understanding of DRUJ mechanics can aid in addressing these concerns.

In terms of joint mechanics, the ulnar fovea, which is situated at the base of the ulnar styloid, acts as the rotating axis at the wrist, while the radial head serves as the longitudinal axis of rotation for pronosupination at the elbow [6]. The stability of these joints relies on a combination of both dynamic and static soft tissue structures. Among the static stabilizing elements are the components forming the TFCC, initially outlined by Werner and Palmer [7]. This intricate structure encompasses the triangular fibrocartilage, resembling a meniscus, along with the palmar and dorsal radioulnar ligaments, the subsheath of the extensor carpi ulnaris (ECU), and the lunotriquetral and ulnolunate ligaments [8]. The TFCC serves several critical functions, including providing a surface for transmitting axial forces to the ulnar carpus, supporting the ulnar carpus through ligamentous connections, and establishing a stable linkage between the distal radius and ulna at the DRUJ.

Ulnar variance, which describes how long the distal ulna is in relation to the distal radius, has been shown to fall within a certain range in the wrists of healthy people who have not been harmed. Typically, this range spans from -0.13 to -0.29 mm, as reported in one study [9]. Furthermore, while moving from complete supination to pronation, the ulnar variance may change by an average of 1.34 mm [10,11].

Patients having malunion of the distal radius often experience a range of discomforts, including weakness, reduced grip strength, restricted forearm and wrist movement, instability of the DRUJ, and pain on the ulnar side of the wrist [12-15]. During examination, these patients may exhibit a noticeable protrusion of the distal ulna due to volar malunion of the distal radius or a wrist deformity caused by the loss of radial inclination. Pain may be provoked during pronation and supination of a wrist that deviates towards the ulnar side (ulnocarpal stress test) [16]. Likewise, discomfort can arise during flexion and extension of a wrist deviated in the ulnar direction (TFCC stress test) [16]. Radiographically, the radius deformity is assessed by evaluating deviations in ulnar variance, volar tilt, normal inclination, and any compensatory deformities in the midcarpal and proximal rows. To determine ulnar variance, a neutral forearm rotation posteroanterior (PA) radiograph is employed and compared to the uninjured side if positive ulnar variance is suspected as a source of ulnar-sided wrist pain. Cystic alterations in the ulnar head, radial aspect of the triquetrum, or ulnar aspect of the lunate may be seen when there is abnormal positive ulnar variance, which is defined as a difference of >3 mm from the unaffected side. Advanced signs are examined in the radiocarpal, DRUJ, and midcarpal joints. Significant health issues might arise for patients with symptomatic malunion of DRFs. Post-traumatic ulnar impaction syndrome, which often develops after a DRF, is one such consequence [17]. Patients typically present with complaints of pain and a notable reduction in grip strength. Prior to considering surgical intervention, it is crucial to conduct a comprehensive clinical and radiological evaluation, with a particular emphasis on determining the patient's functional needs.

There are several surgical treatment options available to address this condition, each tailored to the specific requirements of the patient. These options include corrective procedures such as distal ulnar resection (Darrach's procedure), radial lengthening and ulnar shortening osteotomies, hemi-resection, the Sauvé-Kapandji procedure, interpositional arthroplasty, and DRUJ replacements [18].

There is a limited amount of research available that investigates the functional results of these surgical interventions. Ulnar shortening osteotomies, characterized by their technical simplicity and low incidence of complications, are performed with the goal of averting ulnar-carpal abutment and re-establishing congruity in the DRUJ. In a majority of cases, encouraging functional outcomes have been documented during mid-term follow-up evaluations.

## Conclusions

Ulnar osteotomy stands out as a valuable surgical approach for addressing radius malunion, demonstrating significant enhancements in both radiographic and clinical aspects. This report underscores the efficacy of ulnar osteotomy in the restoration of wrist function, pain alleviation, and the enhancement of patient contentment. It should be duly regarded as a viable choice in the surgical management of radius malunion, presenting the prospect of enhanced outcomes and improved quality of life for individuals grappling with this condition.
